# A set of systematic reviews to help reduce inappropriate prescribing to older people: study protocol

**DOI:** 10.1186/s12877-017-0570-9

**Published:** 2017-10-16

**Authors:** Yolanda V. Martinez, Anna Renom-Guiteras, David Reeves, R. Erandie Ediriweera de Silva, Aneez Esmail, Ilkka Kunnamo, Anja Rieckert, Christina Sommerauer, Andreas Sönnichsen

**Affiliations:** 1 NIHR School for Primary Care Research, Manchester Academic Health Science Centre, University of Manchester, Manchester, England UK; 2 Institute of General Practice and Family Medicine, Witten/Herdecke University, Witten, Germany; 3 Department of Geriatrics, University Hospital Parc de Salut Mar, Barcelona, Spain; 4 Family Medicine Unit, Faculty of Medicine, University of Colombo, Colombo, Sri Lanka; 5 Duodecim Medical Publications Ltd, Helsinki, Finland

**Keywords:** Polypharmacy, Multimorbidity, Older adults, Inappropriate prescribing

## Abstract

**Background:**

Multimorbidity and polypharmacy are common in older people. Assessment tools or lists of criteria aimed at supporting prescription decisions for older people exist, but have often been based on expert opinion with insufficient consideration of the evidence available. The present paper describes the methods we are using to systematically review the existing evidence on the efficacy and safety of the most commonly prescribed drugs for older people in the management of their chronic medical conditions and to develop recommendations to reduce inappropriate prescriptions for incorporation into the Comprehensive Medication Review (CMR) tool developed by the PRIMA-eDS European project.

**Methods:**

We selected the 20 most relevant drugs/drug classes in terms of prescription volumes and risk of hospitalisation for older people and the most relevant indications for the most common chronic conditions among older people and a total of 35 distinct drug-indication pairs were chosen. Based on clinical considerations we collapsed some indications together, reducing the 35 pairs to a final total of 22 separate systematic reviews (SR). A common methodology will be used for each individual SR, based on the methodological manuals of the Cochrane collaboration and the PRISMA statement for reporting systematic reviews. Our search strategy will have a staged approach where we initially search for systematic reviews and meta-analyses, but if relevant reviews are not found, then search for individual studies (controlled intervention and observational studies). Our pilot work and initial scoping of the literature suggested that very few, relevant individual trials or existing systematic reviews have researched or reported exclusively on older people. Therefore, sufficient data might not be available to perform meta-analysis but we will provide a narrative synthesis describing characteristics and findings of included studies. The collected evidence will be used to construct recommendations on when not to use or to discontinue a drug, or when to reduce its dose. Recommendations will be developed in team meetings using the GRADE methodology to reflect the strength of the recommendation and the quality of the evidence. Recommendations will be built into the CMR tool.

**Discussion:**

This protocol has been prepared for a series of systematic reviews which will provide research-based evidence to develop recommendations to reduce inappropriate polypharmacy in older people as part of the CMR tool of the PRIMA-eDS project.

**Electronic supplementary material:**

The online version of this article (doi:10.1186/s12877-017-0570-9) contains supplementary material, which is available to authorized users.

## Background

As a consequence of increasing life-expectancy and medical progress, multimorbidity and its corollary polypharmacy have been increasing in recent years and this is seen most distinctly in older adults [1, 2]. Multimorbidity defined as two or more chronic conditions affects more than half of the population > 75 years of age [3, 4]. Depending on setting between 34% and 59% of people >75 years are exposed to five or more drugs [5–7]. A study of people attending the emergency department at a hospital in London reported that half of the people over 75 years of age were on five or more prescription drugs [5], and another study found that 24% of residents admitted to 57 nursing homes in 8 European countries were prescribed ten or more drugs [8].

The volume of good quality clinical trial evidence for the use of medications in older people is limited as this population group has been often excluded [9, 10]. Thus, although the use of many drugs may be recommended by clinical guidelines, guidelines themselves are often based on evidence from younger populations and older people may not obtain the same benefits [11]. Many of the drugs often prescribed to older people with comorbidity have an important risk of adverse events and hospitalisation in this population [1, 12, 13]. There is therefore a necessity to carefully weigh the risks and benefits of medication in older multimorbid people [14].

Several authors have developed assessment tools or lists of criteria aimed at supporting prescription decisions for older people [15]. However, these assessment tools have often been principally based on expert opinion with insufficient consideration of the evidence available. Some authors have reviewed and narratively synthetized the evidence available on the use of certain drugs with older people. However, to the best of our knowledge no study has systematically reviewed and analysed the benefits and risks of the drugs most commonly used by older people.

PRIMA-eDS is a European Union 7th Framework Programme project (grant agreement No. 305388) across 5 European countries (Austria, Finland, Germany, United Kingdom, Italy) whose main objective is to develop and trial an electronic decision support tool (Comprehensive Medication Review tool [CMR tool]) to reduce polypharmacy in older people with multiple chronic conditions. The present paper describes the methods we are using to systematically review the existing evidence on the efficacy and safety of the most commonly prescribed drugs for older people in the management of their chronic medical conditions and to develop recommendations to reduce inappropriate prescriptions for incorporation into the CMR tool.

## Methods

### Selection of drug classes

From prescribing data taken from national reports and health insurance companies for three of the PRIMA-eDS participating countries (Social Insurance Institute of Finland/Statistical Database Kelasto in Finland (Jaana Harsia-Alatalo, People aged over 75 using prescription drugs, Social Insurance Institute of Finland, personal communication, 2012); Medicines Utilisation Monitoring Centre (OsMed) in Italy [16], research network of the Italian General Practice (Giuliano Piccoliori, The most common drugs in people over 74, personal communication, 2012); and AOK North Rhine-Westphalia in Germany (AOK Northwest Department of Pharmacology, List of most commonly prescribed drugs in people >65 years of age, Dortmund, 2012, unpublished data)), and from a pilot study carried out in Austria [17], we identified seventy five drugs or drug classes as the most commonly prescribed to people aged 65 years and older. In conjunction with this, we conducted a literature search for information about which drugs are most often responsible for hospitalisation due to adverse events in the general population and in older people [12, 18–21]. From this we selected the 20 most relevant drugs or drug classes in terms of prescription volumes and risk of hospitalisation. Some medications can be used for more than one indication and in these cases we included the most relevant indications, giving priority to the most common chronic conditions among older people, i.e. cardiovascular disease (including coronary heart disease, cerebrovascular disease and peripheral vascular disease), heart failure, hypertension, atrial fibrillation, type 2 diabetes mellitus, musculoskeletal disorders including pain, COPD, and mental diseases [22]. We identified a total of 35 distinct drug-indication pairs (Table 1).

**Table 1:** List of drugs and drug classes covered, corresponding indications and SRs performed

Drug or drug class	Indication
Proton pump inhibitors	GERD/GORD (SR-1)
	Gastrointestinal ulcer (gastric, duodenal) (SR-1)
	Dyspepsia (SR-1)
Anti- inflammatory and anti- rheumatic products, non-steroids (NSAIDs)	Musculoskeletal disorders (SR-2)
Opioids	Pain (SR-3)
HMG CoA reductase inhibitors (Statins)	Prevention of cardiovascular disease (SR-4)
Beta blocking agents	Hypertension (SR-5)
	Heart failure (SR-6)
Angiotensin Converting Enzyme inhibitors (ACE-I)	Hypertension (SR-7)
Low-ceiling diuretics, Thiazides and Sulfonamides	Heart failure (SR-8)
	Oedema (SR-8)
	Hypertension (SR-9)
Calcium Channel Blockers (CCBs)	Angina (SR-10)
	Hypertension (SR-11)
Nitrates	Angina (SR-12)
High ceiling diuretics	Heart failure (SR-13)
	Oedema (SR-13)
	Hypertension (SR-13)
	Liver failure (SR-13)
	Renal failure (SR-13)
Platelet aggregation inhibitors	Prevention and treatment of cardiovascular disease including cerebral infarction and transient ischaemic attack, coronary disease and peripheral artery occlusive disease (SR-14)
Vitamin K antagonists (VKA) Direct thrombin inhibitors and Direct factor Xa inhibitors (New Oral Anticoagulants (NOACs))	Deep vein thrombosis/lung embolism (SR-15)
	Thromboembolism in atrial fibrillation (SR-16)
Digitalis glycosides	Heart failure (SR-17)
	Atrial fibrillation (SR-17)
Metformin	Type 2 diabetes (SR-18)
Dipeptidyl peptidase 4 (DPP-4) inhibitors (Gliptins)	Type 2 diabetes (SR-19)
Pioglitazone	Type 2 diabetes (SR-20)
Sulfonylureas	Type 2 diabetes (SR-21)
Glinides	Type 2 diabetes (SR-21)
Insulin and analogues	Type 2 diabetes (SR-22)

*GERD* gastroesophageal reflux disease, *GORD* gastro-oesophageal reflux disease, *HMG CoA* 3-hydroxy-3-methylglutaryl-coenzyme A, *Direct factor Xa inhibitors* xabans

### The systematic review methodology

A separate systematic review (SR) will be conducted for each drug-indication pair. Based on clinical considerations we collapsed some indications together, reducing the 35 pairs to a final total of 22 SRs (Table 1). Each review aims to assess the efficacy and safety of the use of that particular drug with older people in the management of the associated indication or indications. A common methodology will be used for each individual SR, based on the methodological manuals of the Cochrane collaboration [23] and the PRISMA statement for reporting systematic reviews [24].

### Preparatory phase

We developed a Protocol Template (PT) and a Standard Operating Procedures (SOP) document specifying the methodology for the SRs in detail, for reviewers to follow and to achieve uniformity of approach (the PT and SOP can be seen in Additional files 1 and 2). To refine our methodology, the PT and SOP were piloted by two researchers (YVM and ARG) who undertook a SR of the efficacy and safety of metformin in the care of older people with type 2 diabetes.

Next, a team of reviewers competent in English was established and trained on the methods and standard operating procedures by means of 7 workshops delivered by three researchers (YVM, ARG and DR) and other external experts on research methods. Four members of the team will coordinate the SRs (YVM, ARG, CS, and AR). The team of reviewers included professionals with medical background, professionals with methodological background, professionals with both medical and methodological background, professionals with neither medical nor methodological background but involved in the project as study nurse or similar, doctoral, psychology and medical students.

For each SR (i.e. each drug-indication pair), a designated lead reviewer will prepare a topic-specific protocol. This will have the same structure as the PT, but will be adapted appropriately for each drug-indication pair. Furthermore, an unsystematic literature search will be conducted (using PubMed and the Cochrane library) prior to the search in order to identify additional relevant aspects and outcomes to be incorporated in the search and taken into consideration for the risk-to-benefit ratio assessment.

### Study inclusion criteria

#### Types of studies

The SRs will include systematic reviews (including Cochrane reviews), meta-analyses from SRs, controlled interventional studies (e.g. RCTs) and observational studies (i.e. cohort, case-control, cross-sectional, and registry studies).

We will exclude editorials, opinion papers, case reports, case series, narrative reviews, letters, and qualitative studies.

#### Types of participants

Studies with a sufficient number of people ≥65 years old will be included. For Cochrane reviews, systematic reviews and meta-analyses:

- If overall mean or median age is ≥65 years old.
- If overall mean or median age < 65 but a subgroup analysis is provided giving relevant results for people ≥65 years.
- If overall mean or median age is not reported but more than 80% of the studies report a mean or median age ≥ 65 years.

For controlled interventional studies and observational studies:

- If ≥80% of participants are ≥65 years.
- If less than 80% of participants are aged ≥65, but a subgroup analysis is provided giving relevant results for people ≥65 years.

Studies differ in their age inclusion criteria and may report findings for populations or sub-groups defined by different age thresholds than 65, e.g. >70 years. In such cases we will extract and use the results as reported in the study as long as the subgroup is ≥65 years.

#### Types of interventions

Studies will be included if reporting on the efficacy and/or safety of the target chemical substances or drug classes as monotherapy or in combination with any other drug for the treatment of the target indication or indications versus placebo, no treatment, other drugs or a non-pharmacological intervention. We will exclude studies focusing only on acute/short term conditions (e.g. acute management of heart failure or gastrointestinal bleeding in the emergency room).

#### Types of outcomes

We will include any of the following clinically relevant endpoints as primary outcomes for the SRs being conducted:

- Quality of life
- Mortality
- Life expectancy
- Hospitalization
- Cognitive impairment or cognitive status
- Functional impairment or functional status
- Cardiovascular event including stroke
- Renal failure
- Composite end points including any of the above (extraction of individual outcomes will be done if reported by original studies)
- Adverse drug event
- Any of the above evaluated as safety endpoints

Additionally, other relevant outcomes identified for the specific drug classes will be taken into consideration. Outcomes will be extracted in their published data format (e.g. dichotomous, continuous). Due to the fact that we expect a low number of articles to be included for most SRs we will not restrict lengths of follow-up. We will exclude studies evaluating only surrogate endpoints (e.g. blood pressure).

#### Timing

No time limit will be used for searches 1 and 2 but a limit of the last 10 years will be used for search 3B.

#### Setting

Any setting will be included but it should be on the management of chronic conditions. We will not include studies focused on acute treatment.

#### Language

No language restriction will be applied, but we will only include studies that can be read by a member of the research team (English, German, Finnish, Italian and Spanish).

### Search method

The searches will be performed centrally by trained researchers at the University of Manchester (YVM and AW). Our search strategy will have a staged approach where we initially search for systematic reviews and meta-analyses (and stop if an existing review is found, conducted in the previous 2 years, that meets all the objectives of our own review), but if relevant reviews are not found, then search for individual studies (controlled intervention and observational studies). There will be a total of four stages of search, by which each subsequent stage will only be undertaken if the previous one fails to yield high quality results or if the research team decides that the evidence identified so far is not sufficient, or of sufficient quality, to enable an evidence based recommendation to be made.

- Search 1: systematic reviews and meta-analyses in the Cochrane Database of Systematic Reviews (OVID interface, 2005 onwards) and Database of Abstracts or Reviews of Effects (DARE, OVID interface, 1991 onwards).
- Search 2: systematic reviews and meta-analyses in MEDLINE (OVID interface, 1946 onwards), EMBASE (OVID interface, 1974 onwards), Health Technology Assessment (HTA, OVID interface 2001 onwards) and International Pharmaceutical Abstracts (IPA, OVID interface 1970 onwards).
- Search 3A: controlled intervention and observational studies from systematic reviews and meta-analysis not included in searches 1 and 2 but containing eligible studies.
- Search 3B: controlled intervention and observational studies in MEDLINE, EMBASE, HTA and IPA. This search will be limited to the last 10 years.

In addition to database searches, the references of included studies will be checked to obtain a comprehensive list of studies. The citations will be scrutinized and the full manuscripts will be obtained of all citations potentially meeting the inclusion criteria. Study protocols will be also collected to consider for future updates of the SRs. A list of excluded studies after full-text check will be reported for each SR.

The PICOS-framework will be used in the development of the search terms (population, intervention, comparison, outcomes and study design). The following schema for the search terms/MeSH Terms will be used:

1. : Population: “old* adult*”, “old* people”, “geriatric patient*”, elder*, also the MeSH terms “aged” and “frail elderly”
2. : Indication: search terms/MeSH terms for the most frequent chronic conditions related to the target drugs
3. : Drug: search terms/MeSH terms for the target drugs
4. : Outcome: “quality of life”, “mortality”, “life expectancy”, “cardiovascular event”, “hospitalisation”, "hospitalization", “cognitive impairment”, “cognitive status”, “functional status”, “functional impairment”, “renal failure”, “adverse drug event”, “falls” and also the MeSH terms “renal insufficiency”, adverse effects, “drug toxicity”, “patient safety”, “delirium”
5. Study: search terms/MeSH terms for study design. For this theme, we will use specific search terms for both systematic reviews and individual studies. For systematic reviews, we have adapted a search filter from PubMed [25]. MEDLINE is the database used by PubMed but we will also use other databases. Therefore, corresponding search terms will be specified for all databases. For individual studies, we will use the search filters suggested in the Cochrane Handbook for randomised trials [23]. For observational studies, we will use the search filters validated by Fraser in 2006 [26]. The search terms used by MEDLINE and EMBASE can vary and Fraser (2006) also includes specific search filters for each of these databases which we will use as recommended. The full list of search terms can be seen in the example in Additional file 3.

In the search, all terms within a theme will be connected by “OR”, and the five themes will be connected by “AND”. We are using OVID as the search engine to cover all target databases [27].

Additional file 3 shows a complete example of the search done for the SR on metformin for the management of type-2 Diabetes.

### Data management

Literature search results will be uploaded to Endnote X7 reference management software package. Endnote will be used to retrieve search results and to de-duplicate references.

### Selection of studies

Titles and abstracts derived from each search will be assessed by two independent researchers (at least one with a medical background) to identify studies which meet the inclusion criteria. The full manuscripts will be obtained for all titles that appear to meet the inclusion criteria or where there is any uncertainty for inclusion. The reviewers will resolve any disagreements by discussion and, if necessary, by consulting a third reviewer. At the end of each search stage, based on assessment of the quality and quantity of the evidence obtained so far a decision will be made whether or not to proceed to the next stage of search. The list of references to be included for assessment in Search 3A will be prepared by the researchers in parallel to the study selection for Searches 1 and 2.

### Data extraction

One of the reviewers will independently perform data extraction of the included studies using a standardised and piloted data collection form [see Additional file 4] specific to each type of study design (i.e. systematic reviews, controlled interventional studies or observational studies). The second reviewer will check the form and if in their view it is not completely or accurately filled in, will discuss it with the first reviewer and, if necessary, also consult a third reviewer. Data extracted will include the specific drugs and dosages under study, study methods, time to follow-up, characteristics of the participants (e.g. setting, age, comorbidity, functional status and cognitive status), outcomes and results.

### Quality appraisal

Quality appraisal will be done using validated assessment tools for each type of study design. Systematic reviews and meta-analyses will be assessed using a measurement tool for the assessment of systematic reviews (AMSTAR) [28, 29]. Clinical trials will be assessed following the Cochrane Collaboration’s tool for assessing risk of bias addressing the seven specific domains: sequence generation, allocation concealment, blinding of participants and personnel, blinding of outcome assessment, incomplete outcome data, selective outcome reporting and “other issues” [23]. The domain of “other issues” will be answered using a list of specific domains for different trial designs. We will also use the second part of the Cochrane Collaboration’s tool to assign a judgement relating to the risk of bias for each study: “Low”, “High”, or “Unclear” risk of bias [23]. Observational studies will be assessed using a selection of questions extracted from the Critical Appraisal Skills Programme (CASP) [30, 31].

For included systematic reviews or meta-analyses used to develop recommendations, both overall quality and also the quality of each included study will be assessed, except where quality appraisal has been undertaken and reported by the study's own authors.

### Dealing with duplicate and companion publications

All publications from a primary study will be considered for inclusion. We will make clear which publications came from the same study when we report the results of the SRs.

Due to our staged approach, it is quite possible that a publication will be found in a search for individual studies as well as in the previous search for systematic reviews or meta-analyses. Individual studies which are part of an already included systematic review or meta-analysis will be included in their own right if they provide additional relevant evidence that would otherwise be omitted. We will transparently report any such instances with the results of the SRs.

### Data synthesis

Our SRs are focused on people aged 65 or older. Our pilot work and initial scoping of the literature suggested that very few, if any, relevant individual trials or existing systematic reviews have researched or reported exclusively on this group. Hence we anticipate that a significant part of the available evidence will come from subgroup analyses undertaken within studies of much wider age-group populations.

For SRs where we find sufficient numbers of studies that have reported results specific to our target group we will undertake meta-analysis to combine estimates of effect size across these. Clinical heterogeneity (e.g. study differences in populations, interventions and outcomes) will need to be considered and we will only combine effect estimates if (a) the outcome reported is in all essentials the same across the studies; (b) the study designs are the same (e.g. RCTs and observational studies will not be pooled); (c) the included populations, mode of drug delivery and study follow-up times are all reasonably comparable. Statistical heterogeneity will be assessed through calculation of the *I* ^*2*^ heterogeneity index. However, in all cases we will conduct both a fixed-effects and a random-effects meta-analysis, the latter using the DerSimonian method unless counter indicated [32]. We will not base use of the random-effects model on a statistically significant *I* ^*2*^ value, as true heterogeneity is often present even when this test is non-significant [33].

Funnel plots will be used to look for evidence of publication bias and where the number of studies being combined is ten or more we will apply Egger’s test for funnel plot asymmetry [23].

If there is not sufficient data to perform meta-analysis, we will provide a narrative synthesis describing characteristics and findings of included studies. For each SR, we will group studies by specific drugs and indications (if applicable) and by outcomes (which will be further categorised as “risks” and “benefits”). The results for each SR will be summarised in tables for: a) characteristics of included studies (i.e. study design, participants, intervention, comparator, follow-up, and outcomes) and b) summary statistics for each outcome (e.g. odds ratios, relative risks, and hazard ratios). All studies will be included in the narrative synthesis regardless of their quality rating, which will be reported and taken into account when drawing conclusions.

### Identification of references of interest for the development of recommendations

Reviewers will identify additional references which did not fulfil the inclusion criteria of the SRs but which they consider of interest for the development of recommendations. These references may be retrieved either from any of the searches of the SR process or from other sources (i.e. snowballing). References of interest are expected to mostly be studies of younger people but which provide relevant evidence on risks-benefits for a given drug-indication pair; however they may also relate to clinical guidelines or expert consensus. Thus, the identification of these references mostly aims at providing the research team with some evidence on the use of the drugs under study in younger populations.

### Development of recommendations

The studies included and the references considered of interest will be summarised in a document which will be used in team meetings to discuss the recommendations. This document will include: a) relevant information from all selected studies such as study design, target population and sample size, intervention and comparison groups, outcomes, main results, subgroup analysis if applicable; b) results of the quality appraisal of the studies; and c) proposed recommendations to discuss. Initial team meetings will be held by the reviewers of each drug-indication pair, a geriatrician and researcher (ARG) and a senior clinician and researcher (AS). In a later stage the Finish team of editors from Duodecim Medical Publications Ltd. lead by IK will participate and approve the recommendations.

The aim here is to use the overall evidence identified on both risks and benefits of each drug for each indication or indications, to develop recommendations about when not to use or to discontinue the drug or when to reduce its dose. It will be important that this process takes full account of the extent and quality of the identified age-group specific evidence, to avoid over-interpretation and to recognise where the evidence is insufficient to draw any firm conclusions. The following hypothetical situations may happen: 1) the evidence on risks on the use of a medication for older people outweighs the evidence on benefits; in this case a recommendation on the potential discontinuation of the drug will be developed; 2) the evidence on benefits outweighs the evidence on risks; in this case no recommendation on the discontinuation of the drug will be developed; 3) the evidence is insufficient on both risks and benefits; in this case we will take into consideration the evidence on general population and clinical guidelines as identified from the additional references of interest and discuss, for each individual case, whether a recommendation on the potential discontinuation of the drug could be developed where clinicians are drawn attention about the lack of evidence on its use for this age group. Recommendations will be given a strength (weak or strong) and the evidence underlying each recommendation will be given a quality (low, moderate or high) following the GRADE methodology [34–36]. The final recommendations will be worded following a standardised schema according to their strength and the quality of their evidence. Recommendations may have to be put into the context of other drugs which are used for the same indication. Data sources, as well as reasons for upgrading or downgrading the evidence will be transparently reported.

Recommendations will be built into the CMR tool and displayed for general practitioners using the tool if their patients are prescribed the relevant drug for the corresponding indications. Recommendations may be linked to certain conditions or symptoms as well, and should allow the process of shared decision making and the reduction of inappropriate prescribing. The standardised schema to word recommendations can be seen in Table 2.

**Table 2:** Standardised schema to word recommendations

Strength of the recommendation	
• Weak recommendation: It is suggested to discontinue *[include the drug]* for the management of *[include the indication]* because it *[include the wording regarding the quality of the evidence]*	
• Strong recommendation It is recommended to discontinue *[include the drug]* for the management of *[include the indication]* because it *[include the wording regarding the quality of the evidence]*	
Quality of the evidence	
• High quality of evidence: increases/decreases/is associated with …	
• Moderate quality of evidence: appears to increase/decrease/be associated with …	
• Low quality of evidence: may increase/decrease/be associated with …	

General practitioners using the CMR tool will be informed and trained that the recommendations made by the tool do not substitute for careful individual clinical considerations or clinical guidelines, but are only intended to support the clinical decision making process.

## Discussion

This protocol has been prepared for a series of systematic reviews which will provide research-based evidence to develop recommendations to reduce inappropriate polypharmacy in older people as part of the CMR tool of the PRIMA-eDS project. We developed a staged approach methodology for a set of systematic reviews which has the advantage of looking at recently synthesised research-based evidence in searches 1 and 2 (i.e. systematic reviews and meta-analysis) and, only if necessary, evidence from single studies in searches 3A and 3B (i.e. individual controlled intervention and observational studies). One purpose of Search 3A is to ensure that relevant individual studies are not missed by being excluded along with a systematic review or meta-analysis – of which they might be part – that failed to meet our inclusion criteria for synthesised evidence.

Full systematic reviews can be time consuming [37] and we are aware of other methods to retrieve research-based evidence, such as “rapid reviews”. However, we decided to tailor our methodology along “traditional” systematic review lines in order to guarantee good quality of the evidence through use of the manuals from the Cochrane collaboration [23] and the PRISMA statement for reporting systematic reviews [24]. Our staged methodology also enables us to reduce the time needed for complete “traditional” systematic reviews thus making it possible to cover a large number of drugs and indications within the limited time frame of our EU-project.

We believe our approach also overcomes some of the limitations of a rapid review, for which there is a lack of published guidelines or explicit methods [37, 38]. Some rapid reviews do not have clear research questions including participants, interventions, comparisons and outcomes (PICOS) [37]. In addition, the streamlining process of rapid reviews can compromise overall quality, for example, to save time a rapid review might not do a comprehensive search in all relevant databases or it might not have more than one reviewer to select, extract data and appraise the quality of included studies [39].

Our search strategy includes specific terms for older people. This might limit our ability to capture all studies that include evidence on older people. However, we will use a broad range of terms for older people (e.g. old* adult*, aged*, elder*, >65) and search for these terms across multiple fields (title, abstract, and subject headings) in order to reduce the risk that important studies may not be picked up.

We have identified 75 drugs or drug classes as the most commonly prescribed to older people. Although it is not possible to review all of these within the PRIMA-eDS European project, more drugs or drug classes will be reviewed after the first 20 SRs are completed under the project. The Comprehensive Medication Review (CMR) tool is based on drug databases by Duodecim Medical Publications Ltd. and the results of the PRIMA-eDS systematic reviews. Most of the seventy-five drugs/drug classes are covered by the CMR tool with recommendations based on other sources or methods. For example, there are specific recommendations on older people regarding the use of antidepressants, corticosteroids for asthma, methotrexate, among others.

Older people comprise the great majority of consumers of drugs used in the treatment of chronic diseases, yet there has been little previous work to systematically assess the research evidence for usage in this population. The specific approach we have developed and described in this protocol will allow us to conduct a large number of reviews in a systematic and efficient manner but still adhere to recognised standards of quality. The resulting reviews will represent an important step forward in addressing this major gap in knowledge. Through their incorporation in the PRIMA-eDS CMR tool and through other forms of dissemination, we hope that the recommendations that emerge will be of assistance to both general practitioners and policy-makers in reducing unnecessary polypharmacy and inappropriate prescribing in this vulnerable age-group.

## Additional files

Additional file 1: Protocol template. (DOCX 51 kb)

Additional file 2: Standard operating procedure. (DOCX 77 kb)

Additional file 3: Search-SR-metformin-T2D. (DOCX 35 kb)

Additional file 4: Data extraction form. (XLSX 41 kb)

## Abbreviations

AMSTAR: Measurement Tool to Assess Systematic Reviews
CASP: Critical Appraisal Skills Programme
CMR: Comprehensive Medication Review
COPD: Chronic obstructive pulmonary disease
DARE: Database of Abstracts or Reviews of Effects
GRADE: Grading of Recommendations Assessment, Development and Evaluation
HTA: Health Technology Assessment
IPA: International Pharmaceutical Abstracts
OsMed: Medicines Utilisation Monitoring Centre [L’Osservatorio Nazionale sull’Impiego dei Medicinali - Italian]
PICOS: Population, intervention, comparison, outcomes and study design
PRIMA-eDS: Polypharmacy in chronic diseases: Reduction of Inappropriate Medication and Adverse drug events in elderly populations by electronic Decision Support
PRISMA: Preferred Reporting Items for Systematic Reviews and Meta-Analyses
PT: Protocol template
RCTs: Randomised controlled trials
SOP: Standard Operating Procedures
SR: Systematic review
