# Supplementary material for: A set of systematic reviews to help reduce inappropriate prescribing to older people: study protocol
Source: BMC Geriatr. 2017 Oct 16;17(Suppl 1):231. doi: 10.1186/s12877-017-0570-9 (PMC5647557; doi:10.1186/s12877-017-0570-9)
Supplement: Supplementary file 1 — Protocol template. (DOCX 51 kb) [file 12877_2017_570_MOESM1_ESM.docx]

**NOTE:** **Text highlighted in yellow should be changed for each review undertaken:**

- **Drug**
- **Indication**
- **Who undertook review tasks**
- **Date of protocol update**

Efficacy and Patient safety with include drug in the management of include indication in older adults – a systematic review

# Background

Population ageing has been associated with an increase in the prevalence of chronic diseases. The prevalence of chronic comorbidity and multimorbidity is substantial, and affects more than half of the population over 75 years of age ^[^[^1^](#_ENREF_1)^]^. A study among out-patients in Canada, revealed that 50% of the study population had five or more disease conditions ^[^[^2^](#_ENREF_2)^]^. It is also reported that in the US among people aged 65 years and older, 82% of the subjects had one or more chronic diseases and the prevalence increased with advancing age ^[^[^3^](#_ENREF_3)^]^. It is known that about 30% of the population in the UK, and more than half of over 60 year olds, has a long term condition ^[^[^4^](#_ENREF_4)^]^. Majority of the older adults have several long-term conditions ^[^[^5^](#_ENREF_5)^].^ Multimorbidity in older adults has been estimated to range from 55 to 98% with higher mortality, increased disability, a decline of functional status and a lower quality of life ^[^[^6^](#_ENREF_6)^]^. Fortin et al report that there is an inverse association between QOL (Quality of Life) and multimorbidity ^[^[^5^](#_ENREF_5)^]^. Patient safety can be more adversely affected in multimorbidity.

Polypharmacy has been increasing in the recent years and this is seen most distinctly in older adults ^[^[^7^](#_ENREF_7)^]^. It is also reported that polypharmacy, inappropriate prescribing and Adverse Drug Reactions (ADRs) are common in older adults ^[^[^8^](#_ENREF_8)^]^. It is suggested that there is a necessity to carefully weigh the risks and benefits of medication in older multimorbid patients ^[^[^9^](#_ENREF_9)^]^. Ambulatory care is an important setting for healthcare delivery and thus emphasises the importance of patient safety in ambulatory care ^[^[^10^](#_ENREF_10)^]^. A study among Australian GP practices revealed that about two errors are reported for every 1000 individual patients seen by a GP ^[^[^11^](#_ENREF_11)^]^.

Include background for drug and indication.

The balance between risks and benefits of the use of drug in the management of condition in older adults with multimorbidity is not clear.

# Aims

The general objectives of this systematic review are:

- to identify the risks and benefits of the use of drug in the treatment of condition in older adults,
- to provide recommendations when to use and which doses, and when not to use or discontinue drug in the treatment of condition in older adults, and
- to translate these recommendations into algorithms and decision rules to be used in an electronic decision support tool in WP 3 of the PRIMA-eDS-project.

# Methodology

This systematic review will be carried out according to standard protocols for systematic reviews, based on the methodological manuals of the Cochrane collaboration, the Ludwig-Boltzmann Institute for Health Technology Assessment (LBI-HTA, Wien) and the PRISMA statement for reporting systematic reviews. We will first search for existing systematic reviews (i.e. Cochrane reviews). If high quality reviews are available, we will only perform an update search from the date of the systematic review to present. If no high quality systematic review is available we will perform a complete literature search as described below.

## Definitions

We define older adults as people over the age of 65 years**.** Include definition of the indication.

## Inclusion criteria

- Systematic reviews and original studies evaluating benefits and/or risks of drug in the treatment of indication
- Age ≥ 65 years

For systematic reviews:

Those systematic reviews which have included studies focusing on older people:

- - - Included if general mean or median age of the systematic review ≥ 65 years old.
    - Included if general mean or median age <65 but subgroup analysis comparing patients <65 vs. ≥65 years was done and reported. *NOTE: information on whether a subgroup analysis was done has to be checked in the* ***full text****; see advice on how to look for subgroup analysis at the document SOP.*
    - Included if no general mean or median age reported but more than 80% of the studies report a mean or median age ≥65 years; excluded if more than 20% of the studies report a mean or median age <65 years old. However, consider individual studies were mean or median age ≥65 years for Search 3.a.

For original studies:

- - - Included if at least 80% ≥65 years were included. Please always use the calculator (excel document “AGE-CALCULATOR”).
    - Included if the study included less than 80% of participants aged ≥65, but there is a subgroup analysis comparing patients <65 vs. ≥65 years. *NOTE: information on whether a subgroup analysis was done has to be checked only if studies included >100 participants and if the mean or median age was ≥60 years old. It has to be checked in the* ***full text***
- Studies evaluating clinically relevant endpoints as described in table 1.
- The following publication types will be included
  - Cochrane reviews, systematic reviews and meta analyses
  - Controlled interventional studies e.g. randomized controlled trials
  - Observational studies, only if they include a sufficient number of participants and if they provide information of interest regarding adverse events of the medication group under study

If the original study or systematic review focuses on another medication or medication group or non-pharmacological therapy, this article will be excluded unless this intervention is directly compared with the medication under review (in this case drug), and if this information appears at the abstract.

If the original study or systematic review focuses on a combination of two drugs or drug groups including drug, the article will be included if the other inclusion criteria are fulfilled.

## Exclusion criteria

- Studies focusing only on acute/short term conditions
- Studies evaluating only surrogate endpoints (like blood pressure)
- The following publication types will be excluded
  - Editorials
  - Opinion papers
  - Case reports, case series
  - Narrative reviews
  - Letters
  - Qualitative studies
  - Observational studies, if they do not include a sufficient number of participants, neither provide information of interest regarding adverse events

## PICOS Framework

The PICOS-framework to be used in the development of the search terms is depicted in table 1.

Table 1: PICOS-framework

| Population | Older adults with indication |
| --- | --- |
| Intervention | Treatment of indication with drug |
| Control | Treatment of indication with other classes of other drugs or no treatment including placebo |
| Outcome | - quality of life - mortality - life expectancy - hospitalization - cognitive impairment or cognitive status - functional impairment or functional status - cardiovascular event including stroke - renal failure - compound end points including any of the above - adverse drug event - safety |
| Study design | Epidemiological studies including a control group, and controlled interventional studies |

## Search methods

The following databases will be searched: The Cochrane Database of Systematic Reviews (CDSR), The Database of Abstracts of Reviews of Effects (DARE), MEDLINE, EMBASE, Health Technology Assessment Database (INAHTA), International Pharmaceutical Abstracts database (IPA) from the inception of the databases until include month and year.

The search will be limited to studies in humans and older adults (loosely defined, to avoid premature exclusion). The following search terms will be used including variations and combinations.

Search Terms/ MeSH Terms:

Theme 1: Population: “old* adult*”, “old* people”, “geriatric patient*”, elder*, also the MeSH terms “aged” and “frail elderly”

Theme 2: Condition: include search terms/MeSH terms for indication

Theme 3: Drug: include search terms/MeSH terms for drug

Theme 4: Outcome: “quality of life”, “mortality”, “life expectancy”, “cardiovascular event”, “hospitalisation”, “cognitive impairment”, “cognitive status”, “functional status”, “functional impairment”, “renal failure”, “adverse drug event”, “falls” and also the MeSH terms “Renal insufficiency”, adverse effects, “Drug toxicity”, “Patient safety”, “hospitalization”, “Delirium”

In the search, all terms within a theme will be connected by “OR”, and the four themes will be connected by “AND”. Please find the “Search string” in Annex 1.

As described above, the first search (Search 1) will be focused on systematic reviews and meta-analyses using the Cochrane Database of Systematic Reviews and the Database of Abstracts of Reviews of Effects (DARE). If this search identifies studies with a date no older than 2013 and with good quality, no further search will be needed for this drug. If Search 1 does not identify recent and relevant studies of high quality, another search will be performed (Search 2) looking for systematic reviews and meta-analyses using all other databases.

If the previous searches do not identify relevant and recent studies of high quality, controlled intervention studies and observational studies will be searched (Search 3). First, we will check for inclusion all eligible original studies included in the systematic reviews which could not be included in the first and second searches (Search 3.a.). Second, a search will be performed looking for controlled intervention studies and observational studies using all databases, starting with the date of the most recent systematic review existing on this drug and indication (normally it will be an included systematic review, but it may be that it is not included but contains a systematic search on the existing literature) (Search 3.b.). The year in which authors of this systematic review performed their search will be the “cut-off” year for this search. If no systematic review was identified, the search will cover the last 10 years.

Exceptionally, if no systematic review or meta-analysis were included in Search 1 or 2, but a relevant study of good quality is found recommending not using the drug in the general population, reviewers will consider not to continue with the next search, unless reviewers expect that additional studies could change this recommendation for older people.

In addition to database searches, the references of included studies will be checked to obtain a comprehensive list of studies. The citations will be scrutinized and the full manuscripts will be obtained of all citations potentially meeting the inclusion criteria.

## Selection of studies, data extraction and management

Titles and abstracts derived from the first search will be assessed by two independent researchers to identify studies which meet the inclusion criteria. The full manuscripts of the studies relevant to the current study will then be obtained. Review authors will solve any disagreement by discussion and, if necessary, by consulting a third review author.

One of the review authors will independently perform data extraction of the included studies using a standardised and piloted data collection form specific for each study method (i.e. systematic review, clinical trials or observational studies). The second review author will confirm that the form was filled in completely and accurately. If the second review author considers that the form was not completely and/or accurately filled in, he/she should discuss it with the first review author.

Review authors will make a decision whether a second search is needed after data extraction and quality assessment of the studies included within the first search.

Review authors will agree on studies to be included within the second search, perform data extraction and quality assessment using the same procedure as for the first search. Similarly, they will decide whether a third search is necessary and repeat the procedure.

A Standard Operating Procedure (SOP) has been developed in order to guarantee that the review process takes place following the plan established.

## Quality assessment

One of the review authors will independently perform quality appraisal of the included studies using a standardised and piloted quality appraisal form specific for each study method (i.e. systematic review, clinical trials or observational studies). The second review author will confirm that the form was filled in completely and accurately. If the second review author considers that the form was not completely and/or accurately filled in, he/she should discuss it with the first review author. Quality appraisal will be done using validated assessment tools for each study method: Systematic reviews and Meta-analyses will be assessed using the AMSTAR appraisal tool **^[^**[**^12^**](#_ENREF_12)**^]^**; Clinical Trials will be assessed following the Cochrane Handbook for Systematic Reviews of Interventions **^[^**[**^13^**](#_ENREF_13)**^]^**; Observational Studies will be assessed using a selection of questions extracted from the Critical Appraisal Skills Programme (CASP) ^[^[^14^](#_ENREF_14)^,^ [^15^](#_ENREF_15)^]^.

## Grading of the evidence

The quality of the evidence will be assessed by using GRADE methodology ^[^[^16-18^](#_ENREF_16)^]^.

## Transforming evidence into recommendations

After retrieving and grading the evidence we will develop recommendations for the use of drug in older adults with indication which can then be used as decision rules in the PRIMA-eDS-tool. Recommendations will mostly aim at stopping the drug, reconsidering its use, or reducing its dose under certain conditions. Both benefits and risks of the drug will be taken into account when formulating the recommendations.

If there is not sufficient evidence to develop recommendations which can then be used as decision rules in the PRIMA-eDS-tool, clinical guidelines can be used in order to add “recommendations based on guidelines” to the PRIMA-eDS-tool, in order to support our findings or as a complement to our evidence-based recommendations. The decision on which guidelines to be used and whether to use them will be done within the team, and individually for each drug.

**Role of participants**

UNIMAN will perform the database search (YVM/AW). ARG (UWH) and YVM (UNIMAN) will be the reviewers of this systematic review. AS (UWH) will be the 3^rd^ reviewer.

# References

1. Banerjee A, Mbamalu D, Ebrahimi S, Khan AA, Chan TF. The prevalence of polypharmacy in elderly attenders to an emergency department - a problem with a need for an effective solution. Int J Emerg Med. 2011;4(1):22. doi:10.1186/1865-1380-4-22.

2. Fortin M, Hudon C, Dubois MF, Almirall J, Lapointe L, Soubhi H. Comparative assessment of three different indices of multimorbidity for studies on health-related quality of life. Health Qual Life Outcomes. 2005;3:74. doi:10.1186/1477-7525-3-74.

3. Wolff JL, Starfield B, Anderson G. Prevalence, expenditures, and complications of multiple chronic conditions in the elderly. Arch Intern Med. 2002;162(20):2269-76.

4. Department of Health. Long-term conditions. Department of Health, London. 2011. <http://www.dh.gov.uk/en/Healthcare/Longtermconditions/tenthingsyouneedtoknow/index.htm>. Accessed 28/06/2011.

5. Fortin M, Lapointe L, Hudon C, Vanasse A, Ntetu AL, Maltais D. Multimorbidity and quality of life in primary care: a systematic review. Health Qual Life Outcomes. 2004;2:51. doi:10.1186/1477-7525-2-51.

6. Marengoni A, Angleman S, Melis R, Mangialasche F, Karp A, Garmen A et al. Aging with multimorbidity: a systematic review of the literature. Ageing Res Rev. 2011;10(4):430-9. doi:10.1016/j.arr.2011.03.003.

7. Hovstadius B, Hovstadius K, Astrand B, Petersson G. Increasing polypharmacy - an individual-based study of the Swedish population 2005-2008. BMC Clin Pharmacol. 2010;10:16. doi:10.1186/1472-6904-10-16.

8. Schuler J, Duckelmann C, Beindl W, Prinz E, Michalski T, Pichler M. Polypharmacy and inappropriate prescribing in elderly internal-medicine patients in Austria. Wien Klin Wochenschr. 2008;120(23-24):733-41. doi:10.1007/s00508-008-1089-z.

9. Chutka DS, Takahashi PY, Hoel RW. Inappropriate medications for elderly patients. Mayo Clin Proc. 2004;79(1):122-39. doi:10.4065/79.1.122.

10. Hammons T, Piland NF, Small SD, Hatlie MJ, Burstin HR. Ambulatory patient safety. What we know and need to know. J Ambul Care Manage. 2003;26(1):63-82.

11. Makeham MA, Kidd MR, Saltman DC, Mira M, Bridges-Webb C, Cooper C et al. The Threats to Australian Patient Safety (TAPS) study: incidence of reported errors in general practice. Med J Aust. 2006;185(2):95-8.

12. Shea BJ, Grimshaw JM, Wells GA, Boers M, Andersson N, Hamel C et al. Development of AMSTAR: a measurement tool to assess the methodological quality of systematic reviews. BMC Med Res Methodol. 2007;7:10. doi:10.1186/1471-2288-7-10.

13. Higgins JPT, Green S. Cochrane handbook for systematic reviews of interventions. Version 5.1.0 [updated March 2011] The Cochrane Collaboration, 2011. Available from [www.cochrane-handbook.org](http://www.cochrane-handbook.org). editors.

14. Critical Appraisal Skills Programme. 12 questions to help you make sense of cohort study. Oxford. 2013. <http://www.casp-uk.net/wp-content/uploads/2011/11/CASP-Cohort-Study-Checklist-31.05.13.pdf>. Accessed 01/11/2013.

15. Critical Appraisal Skills Programme. 11 questions to help you make sense of case control study. Oxford. 2013. <http://www.casp-uk.net/wp-content/uploads/2011/11/CASP-Case-Control-Study-Checklist-31.05.13.pdf>. Accessed 01/11/2013.

16. Guyatt GH, Oxman AD, Kunz R, Falck-Ytter Y, Vist GE, Liberati A et al. Going from evidence to recommendations. Bmj. 2008;336(7652):1049-51. doi:10.1136/bmj.39493.646875.AE.

17. Guyatt GH, Oxman AD, Kunz R, Vist GE, Falck-Ytter Y, Schünemann HJ et al. What is "quality of evidence" and why is it important to clinicians? Bmj. 2008;336(7651):995-8. doi:10.1136/bmj.39490.551019.BE.

18. Guyatt GH, Oxman AD, Vist GE, Kunz R, Falck-Ytter Y, Alonso-Coello P et al. GRADE: an emerging consensus on rating quality of evidence and strength of recommendations. Bmj. 2008;336(7650):924-6. doi:10.1136/bmj.39489.470347.AD.

19. Fraser C, Murray A, Burr J. Identifying observational studies of surgical interventions in MEDLINE and EMBASE. BMC Med Res Methodol. 2006;6:41. doi:10.1186/1471-2288-6-41.

Prepared by: REED

Date: 20th of March 2013

Updated: July, 15 2013 YVM

Updated: July, 24 2013 YVM and ARG

## Annex 1. Search string

Our primary search will be performed via Ovid EBM Reviews - Cochrane Database of Systematic Reviews and EBM Reviews - Database of Abstracts of Reviews of Effects.

Searches 2 and 3 will be performed via Ovid MEDLINE(R), Ovid MEDLINE(R) In-Process & Other Non-Indexed Citations, EMBASE, Health Technology Assessment, and International Pharmaceutical Abstracts.

We will use the following search string:

**Population**

1. geriatrics.mp. or exp geriatrics/
2. geriatric patient.mp.
3. geriatric*.mp.
4. (elder$ or geriatric$).ab,ti.
5. elder*.mp.
6. frail elderly.mp. or exp frail elderly/
7. aged.mp. or exp Aged/
8. old*.mp.
9. old* adult*.mp.
10. old* people*.mp.
11. >65.mp.
12. over 65.mp.
13. or/1-12

**Condition**

1. include search terms/MeSH terms for indication

**Intervention**

1. include search terms/MeSH terms for drug

**Outcome**

1. mortality.mp. or exp mortality/
2. quality of life.mp. or exp quality of life/
3. QOL.mp.
4. cardiovascular event.mp
5. myocardial infarction.mp
6. stroke.mp.
7. hospitalization.mp. or exp hospitalization/
8. hospitalisation.mp. or exp hospitalisation/
9. life expectancy.mp.
10. cognitive impairment.mp
11. cognitive status.mp.
12. functional status.mp.
13. functional impairment.mp.
14. renal failure.mp.
15. renal insufficiency.mp. or exp renal insufficiency/
16. adverse drug event.mp.
17. adverse effects.mp. or exp adverse effects/
18. drug toxicity.mp. or exp drug toxicity/
19. safety.mp.
20. patient safety.mp. or exp patient safety/
21. falls.mp.
22. delirium.mp. or exp delirium/
23. or/20-41

**Search 1 and 2: Limits, Study designs**

1. (systematic review.ti. or meta-analysis.pt. or meta-analysis.ti. or systematic literature review.ti. or (systematic review.ti,ab. and review.pt.) or consensus development conference.pt. or practice guideline.pt. or cochrane database of systematic reviews.jn. or acp journal club.jn. or health technology assessment winchester england.jn. or evidence report technology assessment summary.jn. or drug class reviews.ti.) or (clinical guideline.tw and management.tw) or ((evidence based.ti. or evidence-based medicine.sh. or best practice*.ti. or evidence synthesis.ti,ab.) and (review.pt. or diseases category.mp. or behaviour.sh. and behavior mechanisms.mp. or therapeutics.sh. or evaluation studies.pt. or validation studies.pt. or guideline.pt. or pmcbook.mp.)) or ((systematic.tw or systematically.tw or critical.ti,ab. or (study selection.tw.) or (predetermined.tw or inclusion.tw and criteri*.tw) or exclusion criteri*.tw. or main outcome measures.tw. or standard of care.tw. or standards of care.tw.) and (survey.ti,ab. or surveys.ti,ab. or overview*.tw. or review.ti,ab. or reviews.ti,ab. or search*.tw. or handsearch.tw. or analysis.ti,ab. or critique.ti,ab. or appraisal.tw. or (reduction.tw. and (risk.sh. or risk.tw.) and (death.mp or recurrence.mp))) and (literature.ti,ab. or articles.ti,ab. or publications.ti,ab. or publication.ti,ab. or bibliography.ti,ab. or bibliographies.ti,ab. or published.ti,ab. or unpublished.tw. or citation.tw. or citations.tw. or database.ti,ab. or internet.ti,ab. or textbooks.ti,ab. or references.tw. or scales.tw. or papers.tw. or datasets.tw. or trials.ti,ab. or meta-analy*.tw. or (clinical.ti,ab. and studies.ti,ab.) or treatment outcome.sh. or treatment outcome.tw. or pmcbook.mp.)) not (letter.pt. or newspaper article.pt. or comment.pt.)

**Search 3: Limits, Study designs**

Search 3 terms from Cochrane ^[^[^13^](#_ENREF_13)^]^ and Fraser (2006) ^[^[^19^](#_ENREF_19)^]^ for MEDLINE

1. randomized controlled trial.pt.
2. controlled clinical trial.pt.
3. randomized.ab.
4. placebo.ab.
5. drug therapy.fs.
6. randomly.ab.
7. trial.ab.
8. groups.ab.
9. or/39-46
10. exp animals/ not humans.sh.
11. 47 not 48
12. Comparative studies/
13. Follow-up studies/
14. Time factors/
15. chang$.tw.
16. evaluat$.tw.
17. reviewed.tw.
18. prospective$.tw.
19. retrospective$.tw.
20. baseline.tw.
21. cohort.tw.
22. case series.tw.
23. or/49-60

Search 3 terms from Cochrane ^[^[^13^](#_ENREF_13)^]^ and Fraser (2006) ^[^[^19^](#_ENREF_19)^]^ for EMBASE

1. random$.mp.
2. factorial$.mp.
3. crossover$.mp.
4. cross over$.mp.
5. cross-over$.mp.
6. placebo$.mp.
7. (doubl$ adj blind$).mp.
8. (singl$ adj blind$).mp.
9. assign$.mp.
10. allocat$.mp.
11. volunteer$.mp.
12. crossover procedure/
13. double blind procedure/
14. randomized controlled trial/
15. single blind procedure/
16. or/39-53
17. Controlled study/
18. Treatment outcome/
19. Major clinical study/
20. Clinical trial/
21. chang$.tw.
22. evaluat$.tw.
23. reviewed.tw.
24. baseline.tw.
25. (compare$ or compara$).tw.
26. or/54-63
