# Supplementary material for: A set of systematic reviews to help reduce inappropriate prescribing to older people: study protocol
Source: BMC Geriatr. 2017 Oct 16;17(Suppl 1):231. doi: 10.1186/s12877-017-0570-9 (PMC5647557; doi:10.1186/s12877-017-0570-9)
Supplement: Supplementary file 2 — Standard operating procedure. (DOCX 77 kb) [file 12877_2017_570_MOESM2_ESM.docx]

**PRIMA-eDS WP-2: Systematic reviews on medication groups**

**Standard Operating Procedure (11.11.13)**

This document is applicable for all medication groups. Inclusion and exclusion criteria can be found in the protocols.

Reviewers will work in teams of 2 (+ a 3^rd^ support reviewer). One reviewer will be chosen who will be responsible for completeness and quality of the whole process. This will be “reviewer 1” and will generally also responsible for:

- Centralising the documents, e.g. filling the excel sheets for “excluded articles” and “protocols”, asking reviewer 3 about opinion, etc.
- Coordinating the search, e.g. contacting the Manchester team for the search, etc.

The other researcher will be “reviewer 2”. Each team could decide how to better organise their work, if they consider.

1. **Preparation of the background (protocol)**

A researcher (normally the reviewer who has medical background) will complete and adapt the document “template of the protocol” to the specific drug and indication under study, including:

- Completing the background section with information which is meaningful for that drug and indication.
- Adding the definition of the condition.
- Adapting the search terms (this task may not be necessary as it may have been done previously)

Other protocols on the same drug or condition could be used as orientation.

Researcher 1 will check the already existing EbMeDS rules on the drug under study in order to check whether there are any studies which may be of interest for the actual systematic review (either by generating work hypothesis or by limiting the length of the search). For example, a study recommending not to use the drug in the general population may help reviewers to decide that only search 1 may be necessary to make recommendations for the old population.

1. **Study selection**

**Comprises different correlative searches (1, 2, 3). After each search reviewers have to make a decision on whether the next search will be necessary and for which time frame, taking the quality and the date of the evidence found into account.**

**Search 1**

- Databases: Cochrane and DARE
- Type of studies: Systematic reviews and meta-analyses*
- Search performed at UniMan (coordinator Yolanda Martínez and Adrine Woodham)
- UniMan prepares an Endnote document containing all the references to be checked for inclusion (titles and abstracts)
- The 2 reviewers (1 and 2) receive the document and each of them checks the references for inclusion according to the predefined inclusion and exclusion criteria (see protocols)**
- Each reviewer enters his/her assessments at the excel document “Selection of studies_*medication group name*” (see example below)

| S**earch 1** | [Reviewer initials] | | | | |
| --- | --- | --- | --- | --- | --- |
|  | Title/abstract check | | | | Full text check |
| Reference | I | U | E | Reasons / comments | Decision (I / U / E) and reason |
| Mayer 2011 |  |  | x |  |  |
| Pérez 2009 |  | X |  |  | Excluded: topic (this article focuses on the treatment of pain with acupuncture and NSAIDs are not considered as comparison group) |
|  |  |  |  |  |  |

- Please insert a cross (“x”) under I (included) or E (excluded) if you can make a decision after reading the title/abstract. If it is helpful for you, you can provide the reasons or comments for this at the column, but this is not obligatory.
- Please insert a cross (“x”) under U (unclear) if you can’t make any decision after reading the title/abstract. Please check the full text and write your final decision and reason(s)*** under “full text check”. If you are unsure about inclusion or exclusion, please report on this and bring this reference to discussion with the other reviewer.
- Full texts which are not available at one university might be available at UniManchester. Please prepare a list and ask Annette Barber or Yolanda Martínez or Adrine Woodham.
- The 2 reviewers make an appointment to discuss their opinions and reviewer 1 develops a joint document. Alternatively, the reviewers can discuss via e-mail. If they disagree in a certain reference, reviewer 3 should help in the final decision.
- All references excluded after full text reading have to be entered at the sheet **“reasons for excluded studies”** of the excel document, and reasons for exclusion proposed at this sheet should be used (i.e. wrong population, wrong intervention, wrong outcome, wrong study design, duplicate, other). The specific details on why this reason was chosen shall be added (for example: wrong outcome, focus on surrogate outcomes (for example, HbA1c for diabetes))

* Published study protocols have to be considered for inclusion (at least to report on them) and those potentially fulfilling the inclusion criteria have to be reported in the fifth worksheet **“Protocols”** at the excel document “Selection of studies_medication group name”.

**this procedure could be done in two different ways (reviewers should agree on which way to be used):

1) the 2 reviewers independently check titles, abstracts and full texts if necessary independently, then reviewer 1 checks were they had any disagreements and they discuss together to make a decision on inclusion. The disadvantage of this way is that the 2 reviewers will more often look for the full texts, but the advantage is that they will not have to read unclear abstracts in two different moments (first abstract and then full text another day).

2) the 2 reviewers independently check only titles and abstracts for inclusion and then reviewer 1 checks which references should be checked in full text –in the case at least one of the reviewers considered a reference “unclear” of “included”, and sends reviewer 2 the full texts; then the two reviewers discuss the full texts for inclusion. The advantage of this way is that full texts will be looked up only once by reviewer 1. The disadvantage is that the 2 reviewers will need to read unclear abstracts/full texts in two different moments.

Reviewers might choose way 1) for Searches 1 and 2 (where there are generally more unclear abstracts) and way 2) for Search 3 (where proportionally less full texts need to be checked).

***Reasons for exclusion given at this column may be **“age group”, “topic”, “method” or “outcome”,** but reviewers can add other reasons if they consider

**After Search 1 reviewers decide whether search 2 is necessary, depending on the quality and date of the systematic reviews found. If good quality reviews can be included dating no older than 2011, Search 2 is not necessary. If a good quality review is included but is older than 2011, Search 2 will be performed with the date of this included review as time limit. Researchers have to inform the Manchester team about this before they perform the search.**

**Search 2**

- Databases: MEDLINE, EMBASE, Health Technology Assessment Database (INAHTA), International Pharmaceutical Abstracts database (IPA)
- Type of studies: Systematic reviews and meta-analyses
- Procedure like for Search 1 (please use the same excel document, worksheet “Search 2”)

**After Search 1 and 2 have been completed, reviewers decide whether search 3 is necessary using the same procedure mentioned above. If search 3 is necessary, both searches 3.A and 3.B will need to be done.**

**Exceptionally, if no systematic review or meta-analysis were included in Search 1 or 2, but a relevant study of good quality is found recommending not using the drug in the general population; reviewers will consider not continuing with the next search, unless reviewers expect that additional studies could change this recommendation for older people.**

**Search 3.A**

Purpose - to identify individual trials and observational studies from searches 1 and 2 which should be considered for inclusion, and to decide whether they should be included.

This search has two parts:

1. Identifying eligible articles.

When examining endnote references for searches 1 and 2 for suitable reviews, we will find some systematic reviews which have to be excluded because they do not fulfill the age criteria, but contain some papers which may fulfill the inclusion criteria for original studies (i.e. clinical trials or observational studies). This could be the case of those articles with mean or median age ≥65. Often, we will find these articles in the tables presented in the systematic reviews. **IMPORTANT NOTE:** Papers with a mean or median age between 60 and 64 are to be considered if they have a sample size bigger than 100.

It is possible that search 1 and 2 also contain original studies (trials and observational studies) and if any of these original studies are eligible for inclusion, they should be included in search 3.A.

Any study that may possibly be suitable should be transferred into sheet 3A of the selection of studies worksheet. The citation details of the study should be entered into the second column “Reference of individual study”. Citation details of the source review should be entered into the first column “Details of source of the study”.

The development of this list “3A” should run in parallel to the process of data selection of searches 1 and 2.

1. Deciding on inclusion.

Once searches 1 and 2 are completed, if reviewers decide that search 3 is needed, then both reviewers should check list 3.A for inclusion, i.e. they should obtain abstracts and if necessary the full text of each article in search 3.A in order to determine suitability for our review (by using the inclusion criteria defined in the study protocol for original studies, i.e. observational studies and clinical trials). Please follow the same procedure for deciding inclusion/exclusion as for searches 1 and 2.

**Search 3.B**

- Databases: MEDLINE, EMBASE, Health Technology Assessment Database (INAHTA), International Pharmaceutical Abstracts database (IPA)
- Type of studies: Controlled intervention studies (Randomized clinical trials, clinical trials) and observational studies (see inclusion criteria)
- **Reviewers should inform the Manchester team who will do the search 3.B on the year in which this search should start. This information will be obtained from the most recent systematic review existing on this drug and indication (normally it will be an included SR, but it may be that it is not included but contains a systematic search on the existing literature). The year in which authors of this SR performed their search will be the “cut-off” year for our new search. If there is not a suitable review, the search should include the last 10 years.**
- Procedure like for Search 1 and 2 (the same excel document can be used, worksheet “Search 3.B”)*

***IMPORTANT NOTE: As Search 3.B. will probably provide a higher number of references to check, reviewers can consider not to copy all authors’ names and years at the excel document. Study selection can be done in the Endnote document by distributing the references to 3 different files: “included”, “unclear”, “excluded”. In this case reviewer 1 should make sure that included articles and unclear articles are entered at the excel document, which will be used as work document for discussing the full texts.**

***IMPORTANT NOTE: For search 3.B., reviewers should make sure that the results are organised by author before they start selecting studies to avoid having a wrong list when they compare/discuss their decisions in selecting articles. As Search 3.B. will probably provide a higher number of references to check, it will be easiest to do the study selection in the Endnote document by distributing the references to 3 different files: “included”, “unclear”, “excluded”. In this case reviewer 1 should make sure that included articles and unclear articles are entered at the excel document, which will be used as work document for discussing the full texts.**

Decision guidance for specific situations during study selection

- If an original study or systematic review (SR) focuses on another medication or medication group or non-pharmacological therapy different from the medication under study, this article will be excluded unless this intervention is directly compared with the medication under study, and ONLY if this information appears at the abstract.
- If the original study or systematic review focuses on a combination of two drugs or drug groups including our medication group of interest, the article will be included if the other inclusion criteria are fulfilled.
- MEAN/MEDIAN AGE: If the abstract does not inform about mean or median age the full text should be checked. The option “search” for words like “age” or “years” or “mean” or “median” is helpful for finding this information.
- SUBGROUP ANALYSIS:
  - If a SR reports on a topic of interest for us, but the general mean or median age is <65 years, we should check in the full text whether a subgroup analysis was done. If the authors do not report on any specific subgroup analysis for people ≥65 years, then we should exclude the SR.
  - For original studies (search 3), if less than 80% of the participants are older than 65 we will check whether a subgroup analysis was performed in the full text, but only if the mean or median age is ≥60 and the sample size is > 100.
  - When checking a full text, the option “search” for words like “age” or “years”, “subgroup”, “geriatric”, “older”, “elder” is helpful for finding the information needed on specific age group analyses.

1. **Putting included articles together and checking reference lists**

- Reviewer 1 will insert all included articles into the excel sheet “Included studies”.
- Once the final list of included articles is clear, one reviewer should check whether in their reference lists are any further articles for inclusion. This reviewer should use the excel sheet “Studies from reference lists” of the excel document and add there:
  - The number of references checked for each included article
  - The references that he/she considers for inclusion, if any.
  - The final decision on inclusion (agreement with second reviewer).
- If any articles are included, their reference lists should be also checked for inclusion using the same procedure.

1. **Quality appraisal and data extraction**

**For Systematic Reviews (SR) (searches 1 and 2)**

- One of the reviewers does data extraction and quality appraisal of all included SRs using:

‘Data extraction and quality appraisal tools.xls’, worksheets:

- - A)SR-Data extr
  - C)SR-Quality appraisal
- The other reviewer confirms the first reviewer’s data abstraction and quality appraisal forms for completeness and accuracy as well as for any disagreement which should be discussed within a meeting. Reviewer 3 helps in case of disagreement.

**For Original Studies (OS) (search 3)**

- Similar procedure, same excel document but different data extraction sheets and quality appraisal tools will be used.
  - For Clinical Trials, worksheets: E)Clinical trials-Data extr and G)Clin Trials Qual appraisal. At worksheet G), columns 1 to 7 should be filled in for all trials. Columns 8 to 18 can be filled in for RCTs if the reviewer considers it is necessary to get a better feeling about the quality of the evidence. Columns 19 to 25 are quality appraisal questions which can be used for specific designs of studies (i.e. Non-randomized controlled trials, Cluster RCT, cross-over trials).
  - For Observational studies, worksheets: I)Observat studies-Data ext and K)Observ studies -Qual apprais

**Data extraction should be done for all included studies but quality appraisal should be done just for included studies to be use in the recommendations which will be decided by the expert team. The last 2 worksheets in the data extraction and quality appraisal Spreadsheet are only for included studies.**

1. **Developing recommendations**

**Study-specific recommendations to stop the drug (to be done by reviewer 1)**

Studies included in the SR comprise both studies reporting on benefits and studies reporting on risks of the drug. Reviewer 1 will:

- Select those included studies which can be used to make recommendations to stop the drug
- Write these recommendations in the corresponding data extraction sheet (specific for each study design)
- Grade the recommendations, specifically for each included article. This means providing information on the QUALITY OF THE EVIDENCE and the STRENGTH OF THE RECOMMENDATION. See sheet F of the excel document “Data extraction and quality appraisal” for further information.
  - For original studies, the grading should be done using the information on quality appraisal and data extraction of each study
  - For systematic reviews, the grading should be done using data extraction of the SR. However, quality appraisal cannot be done using quality appraisal of the SR, but should be done by checking the quality of the individual studies included in this SR. If quality appraisal was done in the SR, this information will be used. If quality appraisal was not done, the reviewers might need to do quality appraisal of the individual studies included.
- Fill out the excel sheet M with all included studies of the SR, their main results, and the corresponding possible stop recommendations for the tool.

The following aspects should also be considered for the development of the recommendations:

- Studies with duration of 6 months or less are not a good source of evidence for the development of recommendations on long-term outcomes such as mortality, but studies with duration of 6 months can be a good source of evidence for the development of recommendations on short-term outcomes such as safety outcomes.
- The minimum duration of a study allowing to develop any recommendations on mortality is around 1.5 years.

If no evidence has been found supporting the use of discontinuation of that drug for that indication, reviewers may search for 1 or 2 clinical guidelines or expert consensus which provide some information on the use of that drug for that indication in older adults. Found guidelines and their results / possible recommendations should be added at the work sheet M. Guidelines which can be checked are: NICE guidelines, expert consensus, other (search individually for each indication and drug).

Studies identified among the EbM guidelines which bring information of interest for the development of the final recommendations should also be added at sheet M.

Other studies on general population of interest identified during the search process should be also added at sheet M.

**General/Final recommendations**

Sheet M will be used for the expert meeting, where a final decision on the recommendations for that drug and indication will be made. These final recommendations may have to be put into the context of other drugs which are used for the same indication.

Recommendations will mostly aim at stopping the drug, reconsidering its use, or reducing its dose under certain conditions. Both benefits and risks of the drug will be taken into account when formulating the recommendations.

Depending on the possible scenarios found, individual decisions will be made for each drug depending on its characteristics. For example:

- If there is good evidence that the drug is not recommended in the general population (adverse events are more important than benefits), most probably a recommendation to stop the drug in older people can be done.
- If there is good evidence that the drug is clearly recommended in the general population, most probably no recommendation to stop the drug in older people can be done.
- If there is no evidence that the drug has any benefits in the general or young population, possibly a recommendation to stop the drug in older people can be done.
- If there is some evidence that the drug has benefits in the general or young population, but no evidence in the older population, a recommendation to consider to stop the drug may be done, depending on the outcome(s) under study. For example, the main outcome of certain drugs may be reduction of mortality (e.g. metformin, statins), while for other drugs it may be symptomatic relieve (e.g. laxatives, antihistaminic drugs). Therefore:
- If the main outcome is reduction of mortality, if we do not find any evidence in the older population but there is some evidence in the younger population, it is quite possible that the benefit of this drug in the older comorbid population is lower than in the younger population because older people have a lower life expectancy and other diseases and polypharmacy, so the deaths attributable to that specific condition or problem are lower, so the treatment effect of that drug for that condition is lower.
- If the main outcome is symptomatic improvement, if we do not find any evidence in the older population but there is some evidence in the younger population, these results may be more appropriately applied to older patients.

Sheet N will be filled out after the expert meeting. It will be the work document used to insert the recommendations into the PRIMA-eDS tool. This will be done by the reviewer team with the support of the Finnish team, who will review the recommendations.

Recommendations should:

- approach “potentially clinically meaningful outcomes”, for example mortality, risk of falls, etc;
- be “condition” oriented, for example “if more than 80 years old”, if “high dependence for ADL”, if “high level of frailty”, if “cognitive impairment”, etc;
- be written allowing GPs to provide information which is meaningful for the patients (e.g. numbers needed to treat rather than relative ratios), allowing shared decision making;
- provide links to the publications for those GPs who wish to check them;
- be activated if the patient presents a specific adverse event. For example if we find a recommendation (either in a guideline or in a study) saying that metformin is associated with nausea, if the patient shows nausea at the follow-up this alarm should be activated;

**ZOTERO**

An updated excel document including the Overview of the reviews is available in ZOTERO (Group libraries ⮧ Prima-eDS ⮧ 1^st^ review: on drug treatment of chronic disease ⮧ Overview of reviews) where reviewers see the actual progression in their reviews as they go along (worksheet “Overview of SR medications”). Reviewer 1 of each search can update this document. When doing so, reviewers should save it with the date of the update and the time and with the initials of the person who modified it. E.g. “Overview of reviews.11.11.13-10.09_AR”

**Basic schema for selection of studies**

List of references

Third reviewer

Include

Exclude (topic /method do not fit or the medication is not mentioned at the abstract or title)

Exclude (mean or median age of the SR <65 years old or no subgroup analyses for older persons)

Unclear

Read full text

Unclear

Read title and abstract

Include
