# Supplementary material for: A set of systematic reviews to help reduce inappropriate prescribing to older people: study protocol
Source: BMC Geriatr. 2017 Oct 16;17(Suppl 1):231. doi: 10.1186/s12877-017-0570-9 (PMC5647557; doi:10.1186/s12877-017-0570-9)
Supplement: Supplementary file 3 — Search-SR-metformin-T2D. (DOCX 35 kb) [file 12877_2017_570_MOESM3_ESM.docx]

**Search 1 and 2: Metformin for the management of type 2 diabetes**

**Databases:**

**EBM Reviews - Cochrane Database of Systematic Reviews [search 1]**

**EBM Reviews - Database of Abstracts of Reviews of Effects [search 1]**

**Ovid MEDLINE(R) [search 2]**

**Ovid MEDLINE(R) In-Process & Other Non-Indexed Citations [search 2]**

**Embase [search 2]**

**EBM Reviews - Health Technology Assessment [search 2]**

**International Pharmaceutical Abstracts [search 2]**

| **#** | **Searches** | **Results** |
| --- | --- | --- |
| **Population** | | |
|  | geriatrics.mp. or exp geriatrics/ |  |
|  | geriatric patient.mp. |  |
|  | geriatric*.mp. |  |
|  | (elder$ or geriatric$).ab,ti. |  |
|  | elder*.mp. |  |
|  | frail elderly.mp. or exp frail elderly/ |  |
|  | aged.mp. or exp Aged/ |  |
|  | old*.mp. |  |
|  | old* adult*.mp. |  |
|  | old* people*.mp. |  |
|  | >65.mp. |  |
|  | over 65.mp. |  |
|  | or/1-12 |  |
| **Condition** | | |
|  | Diabetes Mellitus, Type 2.mp. or exp Diabetes Mellitus, Type 2/ |  |
|  | (MODY or NIDDM or T2DM).tw,ot. |  |
|  | (non insulin$ depend$ or noninsulin$ depend$ or noninsulin?depend$ or noninsulin?depend).tw,ot. |  |
|  | ((typ$ 2 or typ$ II) adj3 diabet$).tw,ot. |  |
|  | ((keto?resist$ or non?keto$) adj6 diabet$).tw,ot. |  |
|  | (((late or adult$ or matur$ or slow or stabl$) adj3 onset) and diabet$).ab,ti. |  |
|  | or/14-19 |  |
|  | Diabetes Insipidus.mp. or exp Diabetes Insipidus/ |  |
|  | diabet$ insipidus.tw,ot. |  |
|  | 21 or 22 |  |
|  | 20 not 23 |  |
| **Intervention** | | |
|  | biguanide*.mp. |  |
|  | metformin.mp. or exp metformin/ |  |
|  | or/25-26 |  |
| **Outcome** | | |
|  | mortality.mp. or exp mortality/ |  |
|  | quality of life.mp. or exp quality of life/ |  |
|  | QOL.mp |  |
|  | cardiovascular event.mp |  |
|  | myocardial infarction.mp |  |
|  | stroke.mp |  |
|  | hospitalization.mp. or exp hospitalization/ |  |
|  | hospitalisation.mp. or exp hospitalisation/ |  |
|  | life expectancy.mp |  |
|  | cognitive impairment.mp |  |
|  | cognitive status.mp. |  |
|  | functional status.mp. |  |
|  | functional impairment.mp. |  |
|  | renal failure.mp |  |
|  | renal insufficiency.mp. or exp renal insufficiency/ |  |
|  | adverse drug event.mp |  |
|  | adverse effects.mp. or exp adverse effects/ |  |
|  | drug toxicity.mp. or exp drug toxicity/ |  |
|  | safety.mp |  |
|  | patient safety.mp. or exp patient safety/ |  |
|  | falls.mp. |  |
|  | delirium.mp. or exp delirium/ |  |
|  | or/28-49 |  |
| **Limits, Study designs** | | |
|  | (systematic review.ti. or meta-analysis.pt. or meta-analysis.ti. or systematic literature review.ti. or (systematic review.ti,ab. and review.pt.) or consensus development conference.pt. or practice guideline.pt. or cochrane database of systematic reviews.jn. or acp journal club.jn. or health technology assessment winchester england.jn. or evidence report technology assessment summary.jn. or drug class reviews.ti.) or (clinical guideline.tw and management.tw) or ((evidence based.ti. or evidence-based medicine.sh. or best practice*.ti. or evidence synthesis.ti,ab.) and (review.pt. or diseases category.mp. or behaviour.sh. and behavior mechanisms.mp. or therapeutics.sh. or evaluation studies.pt. or validation studies.pt. or guideline.pt. or pmcbook.mp.)) or ((systematic.tw or systematically.tw or critical.ti,ab. or (study selection.tw.) or (predetermined.tw or inclusion.tw and criteri*.tw) or exclusion criteri*.tw. or main outcome measures.tw. or standard of care.tw. or standards of care.tw.) and (survey.ti,ab. or surveys.ti,ab. or overview*.tw. or review.ti,ab. or reviews.ti,ab. or search*.tw. or handsearch.tw. or analysis.ti,ab. or critique.ti,ab. or appraisal.tw. or (reduction.tw. and (risk.sh. or risk.tw.) and (death.mp or recurrence.mp))) and (literature.ti,ab. or articles.ti,ab. or publications.ti,ab. or publication.ti,ab. or bibliography.ti,ab. or bibliographies.ti,ab. or published.ti,ab. or unpublished.tw. or citation.tw. or citations.tw. or database.ti,ab. or internet.ti,ab. or textbooks.ti,ab. or references.tw. or scales.tw. or papers.tw. or datasets.tw. or trials.ti,ab. or meta-analy*.tw. or (clinical.ti,ab. and studies.ti,ab.) or treatment outcome.sh. or treatment outcome.tw. or pmcbook.mp.)) not (letter.pt. or newspaper article.pt. or comment.pt.) |  |
| **Population AND Condition AND Intervention AND Outcome AND Limits, Study designs** | | |
|  | 13 and 24 and 27 and 50 and 51 |  |

**Search 3B: Metformin for the management of type 2 diabetes**

**Databases:**

**Ovid MEDLINE(R)**

**Ovid MEDLINE(R) In-Process & Other Non-Indexed Citations**

**EBM Reviews - Health Technology Assessment**

**International Pharmaceutical Abstracts**

| **#** | **Searches** | | **Results** |  |  |
| --- | --- | --- | --- | --- | --- |
| **Population** | | | |  |  |
|  | geriatrics.mp. or exp geriatrics/ |  | |  |  |
|  | geriatric patient.mp. |  | |  |  |
|  | geriatric*.mp. |  | |  |  |
|  | (elder$ or geriatric$).ab,ti. |  | |  |  |
|  | elder*.mp. |  | |  |  |
|  | frail elderly.mp. or exp frail elderly/ |  | |  |  |
|  | aged.mp. or exp Aged/ |  | |  |  |
|  | old*.mp. |  | |  |  |
|  | old* adult*.mp. |  | |  |  |
|  | old* people*.mp. |  | |  |  |
|  | >65.mp. |  | |  |  |
|  | over 65.mp. |  | |  |  |
|  | or/1-12 |  | |  |  |
| **Condition** | | | |  |  |
|  | Diabetes Mellitus, Type 2.mp. or exp Diabetes Mellitus, Type 2/ |  | |  |  |
|  | (MODY or NIDDM or T2DM).tw,ot. |  | |  |  |
|  | (non insulin$ depend$ or noninsulin$ depend$ or noninsulin?depend$ or noninsulin?depend).tw,ot. |  | |  |  |
|  | ((typ$ 2 or typ$ II) adj3 diabet$).tw,ot. |  | |  |  |
|  | ((keto?resist$ or non?keto$) adj6 diabet$).tw,ot. |  | |  |  |
|  | (((late or adult$ or matur$ or slow or stabl$) adj3 onset) and diabet$).ab,ti. |  | |  |  |
|  | or/14-19 |  | |  |  |
|  | Diabetes Insipidus.mp. or exp Diabetes Insipidus/ |  | |  |  |
|  | diabet$ insipidus.tw,ot. |  | |  |  |
|  | 21 or 22 |  | |  |  |
|  | 20 not 23 |  | |  |  |
| **Intervention** | | | |  |  |
|  | biguanide*.mp. | |  |  |  |
|  | metformin.mp. or exp metformin/ | |  |  |  |
|  | or/25-26 | |  |  |  |
| **Outcome** | | | |  |  |
|  | mortality.mp. or exp mortality/ | |  |  |  |
|  | quality of life.mp. or exp quality of life/ | |  |  |  |
|  | QOL.mp | |  |  |  |
|  | cardiovascular event.mp | |  |  |  |
|  | myocardial infarction.mp | |  |  |  |
|  | stroke.mp | |  |  |  |
|  | hospitalization.mp. or exp hospitalization/ | |  |  |  |
|  | hospitalisation.mp. or exp hospitalisation/ | |  |  |  |
|  | life expectancy.mp | |  |  |  |
|  | cognitive impairment.mp | |  |  |  |
|  | cognitive status.mp. | |  |  |  |
|  | functional status.mp. | |  |  |  |
|  | functional impairment.mp. | |  |  |  |
|  | renal failure.mp | |  |  |  |
|  | renal insufficiency.mp. or exp renal insufficiency/ | |  |  |  |
|  | adverse drug event.mp | |  |  |  |
|  | adverse effects.mp. or exp adverse effects/ | |  |  |  |
|  | drug toxicity.mp. or exp drug toxicity/ | |  |  |  |
|  | safety.mp | |  |  |  |
|  | patient safety.mp. or exp patient safety/ | |  |  |  |
|  | falls.mp. | |  |  |  |
|  | delirium.mp. or exp delirium/ | |  |  |  |
|  | or/28-49 | |  |  |  |
| **Limits, Study designs** | | | |  |  |
|  | randomized controlled trial.pt. | |  |  |  |
|  | controlled clinical trial.pt. | |  |  |  |
|  | randomized.ab. | |  |  |  |
|  | placebo.ab. | |  |  |  |
|  | drug therapy.fs. | |  |  |  |
|  | randomly.ab. | |  |  |  |
|  | trial.ab. | |  |  |  |
|  | groups.ab. | |  |  |  |
|  | or/51-58 | |  |  |  |
|  | exp animals/ not humans.sh. | |  |  |  |
|  | 59 not 60 | |  |  |  |
|  | Comparative studies/ | |  |  |  |
|  | Follow-up studies/ | |  |  |  |
|  | Time factors/ | |  |  |  |
|  | chang$.tw. | |  |  |  |
|  | evaluat$.tw. | |  |  |  |
|  | reviewed.tw. | |  |  |  |
|  | prospective$.tw. | |  |  |  |
|  | retrospective$.tw. | |  |  |  |
|  | baseline.tw. | |  |  |  |
|  | cohort.tw. | |  |  |  |
|  | case series.tw. | |  |  |  |
|  | or/61-72 | |  |  |  |
| **Population AND Condition AND Intervention AND Outcome AND Limits, Study designs** | | | |  |  |
|  | 13 and 24 and 27 and 50 and 73 | |  |  |  |

**Search 3B: Metformin for the management of type 2 diabetes**

**Database:**

**Embase**

| [**#**](http://ovidsp.uk.ovid.com/sp-3.8.1a/ovidweb.cgi?&S=DCDLPDGALHHFOEDMFNOKEBEGNLKJAA00&Sort+Sets=descending) | **Searches** | **Results** |
| --- | --- | --- |
| **Population** | | |
|  | geriatrics.mp. or exp geriatrics/ |  |
|  | geriatric patient.mp. |  |
|  | geriatric*.mp. |  |
|  | (elder$ or geriatric$).ab,ti. |  |
|  | elder*.mp. |  |
|  | frail elderly.mp. or exp frail elderly/ |  |
|  | aged.mp. or exp Aged/ |  |
|  | old*.mp. |  |
|  | old* adult*.mp. |  |
|  | old* people*.mp. |  |
|  | >65.mp. |  |
|  | over 65.mp. |  |
|  | or/1-12 |  |
| **Condition** | | |
|  | Diabetes Mellitus, Type 2.mp. or exp Diabetes Mellitus, Type 2/ |  |
|  | (MODY or NIDDM or T2DM).tw,ot. |  |
|  | (non insulin$ depend$ or noninsulin$ depend$ or noninsulin?depend$ or noninsulin?depend).tw,ot. |  |
|  | ((typ$ 2 or typ$ II) adj3 diabet$).tw,ot. |  |
|  | ((keto?resist$ or non?keto$) adj6 diabet$).tw,ot. |  |
|  | (((late or adult$ or matur$ or slow or stabl$) adj3 onset) and diabet$).ab,ti. |  |
|  | or/14-19 |  |
|  | Diabetes Insipidus.mp. or exp Diabetes Insipidus/ |  |
|  | diabet$ insipidus.tw,ot. |  |
|  | 21 or 22 |  |
|  | 20 not 23 |  |
| **Intervention** | | |
|  | biguanide*.mp. |  |
|  | metformin.mp. or exp metformin/ |  |
|  | or/25-26 |  |
| **Outcome** | | |
|  | mortality.mp. or exp mortality/ |  |
|  | quality of life.mp. or exp quality of life/ |  |
|  | QOL.mp |  |
|  | cardiovascular event.mp |  |
|  | myocardial infarction.mp |  |
|  | stroke.mp |  |
|  | hospitalization.mp. or exp hospitalization/ |  |
|  | hospitalisation.mp. or exp hospitalisation/ |  |
|  | life expectancy.mp |  |
|  | cognitive impairment.mp |  |
|  | cognitive status.mp. |  |
|  | functional status.mp. |  |
|  | functional impairment.mp. |  |
|  | renal failure.mp |  |
|  | renal insufficiency.mp. or exp renal insufficiency/ |  |
|  | adverse drug event.mp |  |
|  | adverse effects.mp. or exp adverse effects/ |  |
|  | drug toxicity.mp. or exp drug toxicity/ |  |
|  | safety.mp |  |
|  | patient safety.mp. or exp patient safety/ |  |
|  | falls.mp. |  |
|  | delirium.mp. or exp delirium/ |  |
|  | or/28-49 |  |
| **Limits, Study designs** | | |
|  | random$.mp. |  |
|  | factorial$.mp. |  |
|  | crossover$.mp. |  |
|  | cross over$.mp. |  |
|  | cross-over$.mp. |  |
|  | placebo$.mp. |  |
|  | (doubl$ adj blind$).mp. |  |
|  | (singl$ adj blind$).mp. |  |
|  | assign$.mp. |  |
|  | allocat$.mp. |  |
|  | volunteer$.mp. |  |
|  | crossover procedure/ |  |
|  | double blind procedure/ |  |
|  | randomized controlled trial/ |  |
|  | single blind procedure/ |  |
|  | or/51-65 |  |
|  | Controlled study/ |  |
|  | Treatment outcome/ |  |
|  | Major clinical study/ |  |
|  | Clinical trial/ |  |
|  | chang$.tw. |  |
|  | evaluat$.tw. |  |
|  | reviewed.tw. |  |
|  | baseline.tw. |  |
|  | (compare$ or compara$).tw. |  |
|  | or/66-75 |  |
| **Population AND Condition AND Intervention AND Outcome AND Limits, Study designs** | | |
|  | 13 and 24 and 27 and 50 and 76 |  |
